# Supplementary material for: Estrogenic Effects of Extracts and Isolated Compounds from Belowground and Aerial Parts of Spartina anglica
Source: Mar Drugs. 2021 Apr 11;19(4):210. doi: 10.3390/md19040210 (PMC8069246; doi:10.3390/md19040210)
Supplement: Supplementary file 1 [file marinedrugs-19-00210-s001.pdf]

Estrogenic Effects of Extracts and Isolated Compounds from Belowground and Aerial Parts of *Spartina anglica*

Sullim Lee[1,†], Geum Jin Kim[2,3,†], Hyukbean Kwon[2], Joo-Won Nam[2], Ji Yun Baek[4,5], Sang Hee Shim[6], Hyukjae Choi[2,3*] and Ki Sung Kang[4,*]

[1] Department of Life Science, College of Bio-Nano Technology, Gachon University, Seongnam 13120, Republic of Korea; sullimlee@gachon.ac.kr (S.L.)
[2] College of Pharmacy, Yeungnam University, Gyeongsan 38541, Republic of Korea; canta87@ynu.ac.kr (G.J.K.), zero9602@gmail.com (H.K.), jwnam@yu.ac.kr (J.-W.N.)
[3] Research Institute of Cell Culture, Yeungnam University, Gyeongsan, Gyeongbuk 38541, Republic of Korea
[4] College of Korean Medicine, Gachon University, Seongnam 13120, Republic of Korea; wldbsttn@naver.com (J.Y.B.), kkang@gachon.ac.kr (K.S.K.)
[5] Department of Food Science, Gyeongnam National University of Science and Technology, Jinju 52725 Republic of Korea; wldbsttn@naver.com (J.Y.B.)
[6] Natural Products Research Institute, College of Pharmacy, Seoul National University, 1 Gwanak-ro, Gwanak-gu, Seoul, 08826, Republic of Korea; sanghee_shim@snu.ac.kr (S. H. S.)
* Correspondence: E-mail: kkang@gachon.ac.kr; Tel.: 82-31-750-5402 (K.S.K.), h5choi@yu.ac.kr; Tel.: 82-53-810-2824 (H.C.)
† These authors contributed equally to this work.

Contents

Figure S1. 1D NMR spectra of compound **1** in CD_3OD
Figure S2. 2D NMR spectra of compound **1** in CD_3OD
Figure S3. 1D NMR spectra of compound **2** in DMSO-d_6
Figure S4. 1D NMR spectra of compound **3** in CD_3OD
Figure S5. 1D NMR spectra of compound **4** in CD_3OD
Figure S6. 1D NMR spectra of compound **5** in CD_3OD

Figure S1. 1D NMR spectra of compound **1** in CD_3OD

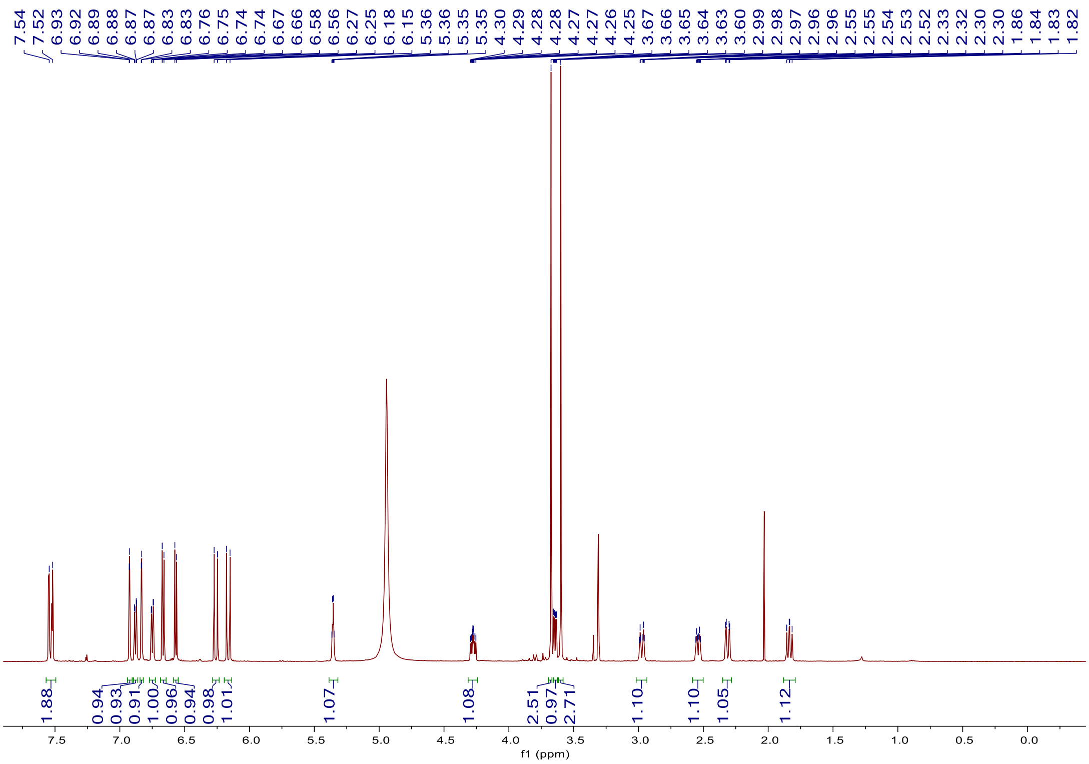

The ^1H NMR spectrum of **1** (600 MHz)

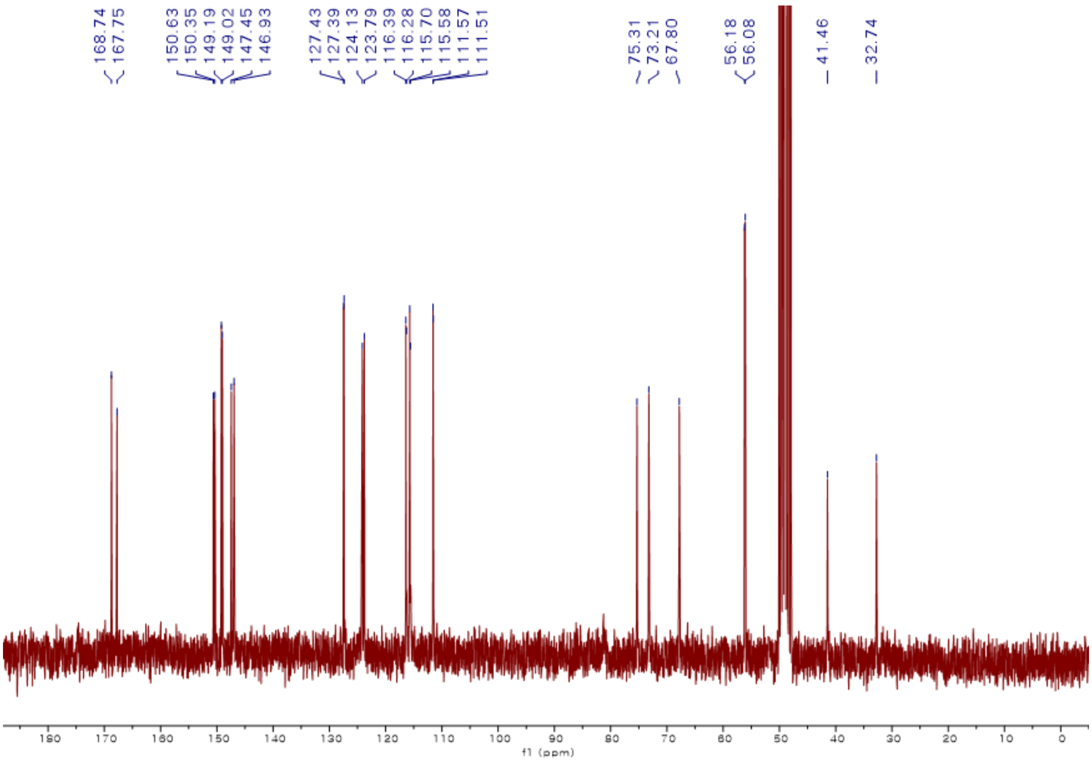

The ^{13}C NMR spectrum of **1** (62.5 MHz)

Figure S2. 2D NMR spectra of compound **1** in CD_3OD

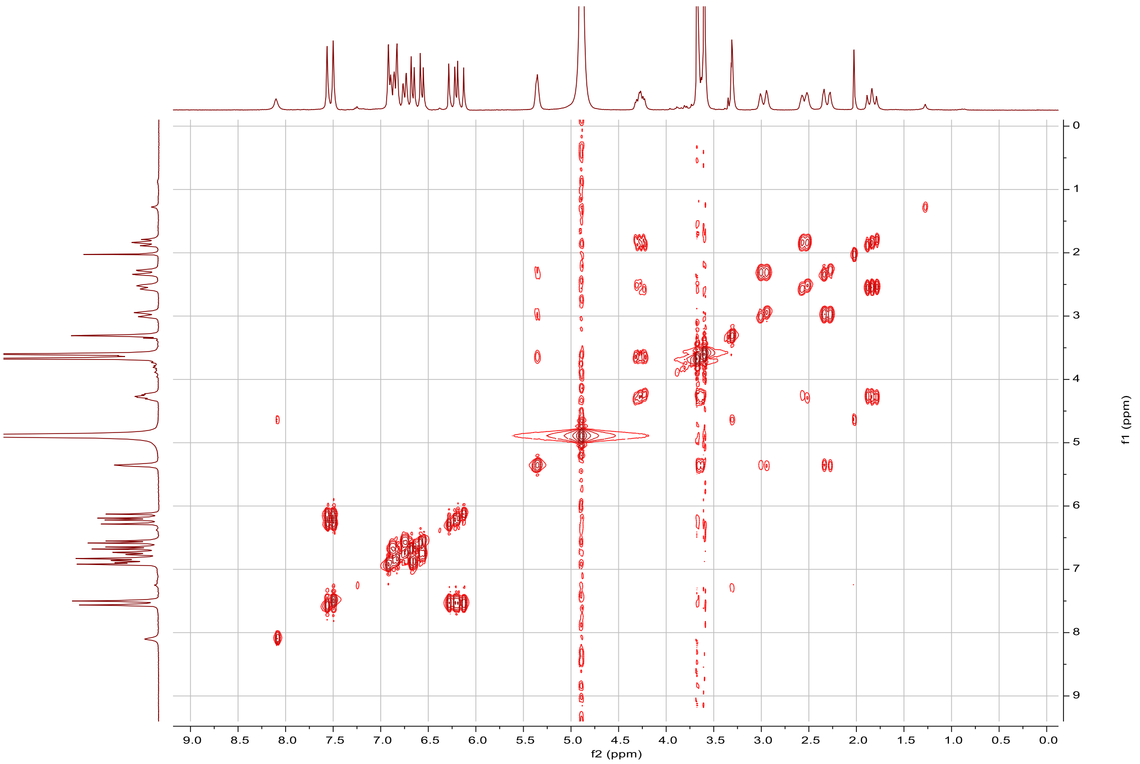

The ^1H-^1H COSY spectrum of **1** (250 MHz)

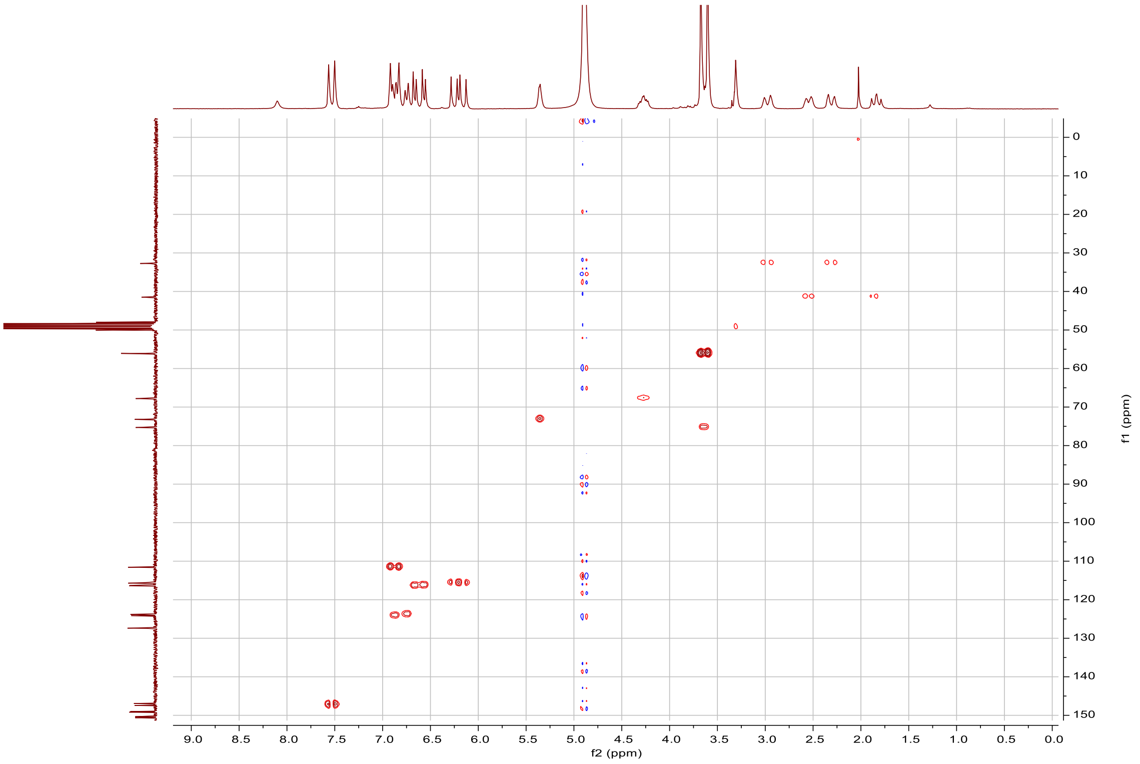

The HMQC spectrum of **1** (250 MHz)

Figure S2. (continued)

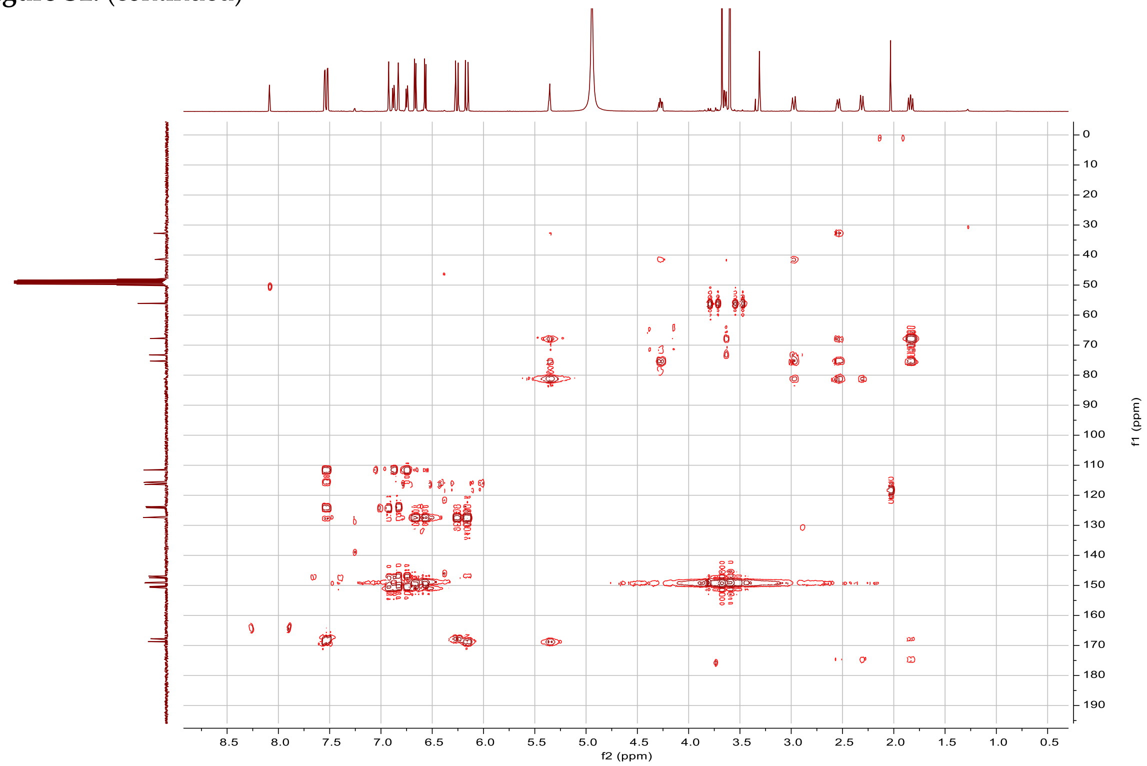

The HMBC spectrum of **1** (600 MHz)

Figure S3. 1D NMR spectra of compound **2** in DMSO-*d*6

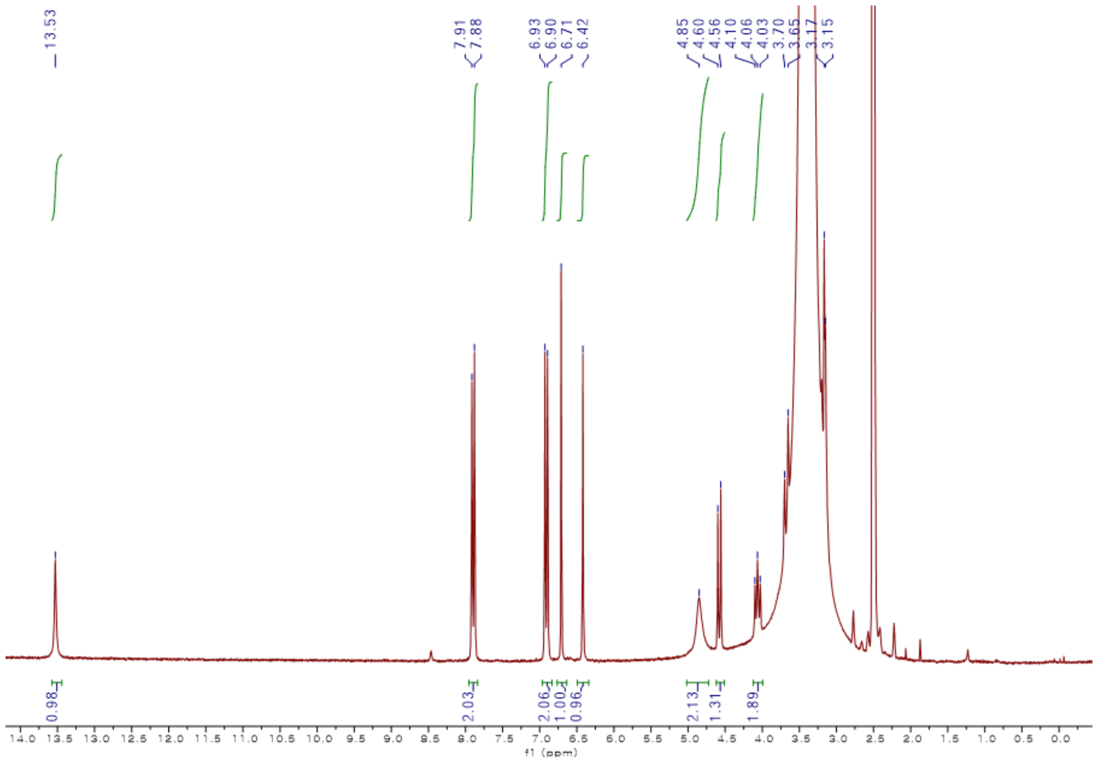

The ^{1}H NMR spectrum of **2** (250 MHz)

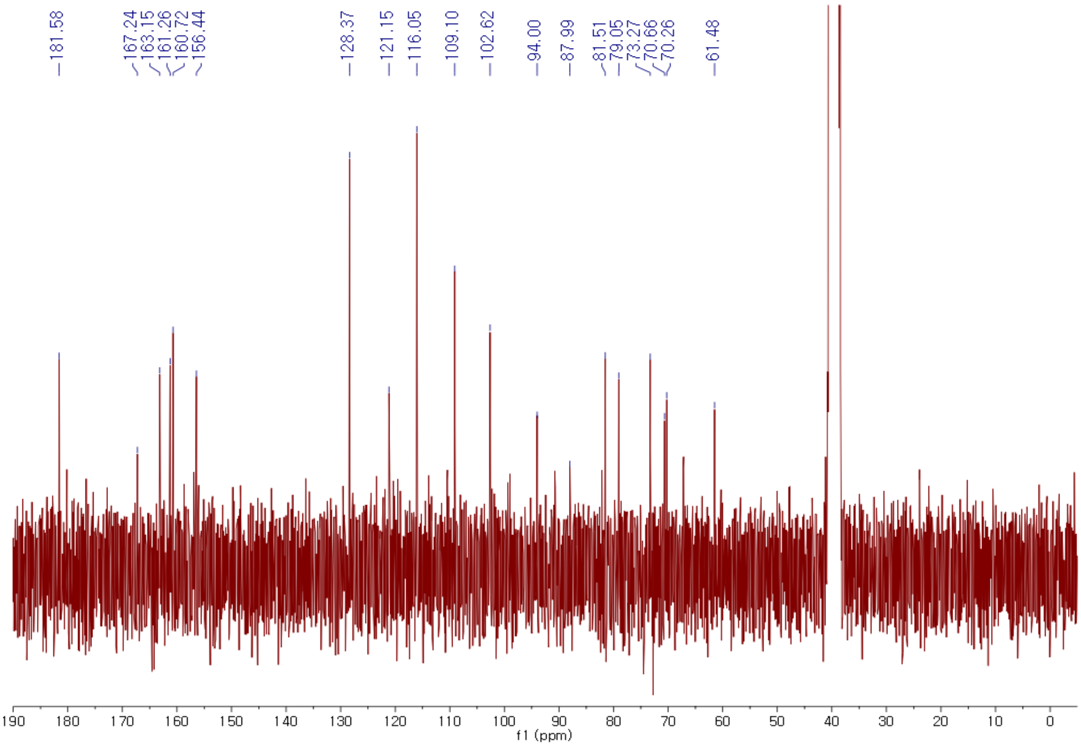

The ^{13}C NMR spectrum of **2** (62.5 MHz)

Figure S4. 1D NMR spectra of compound **3** in CD_3OD

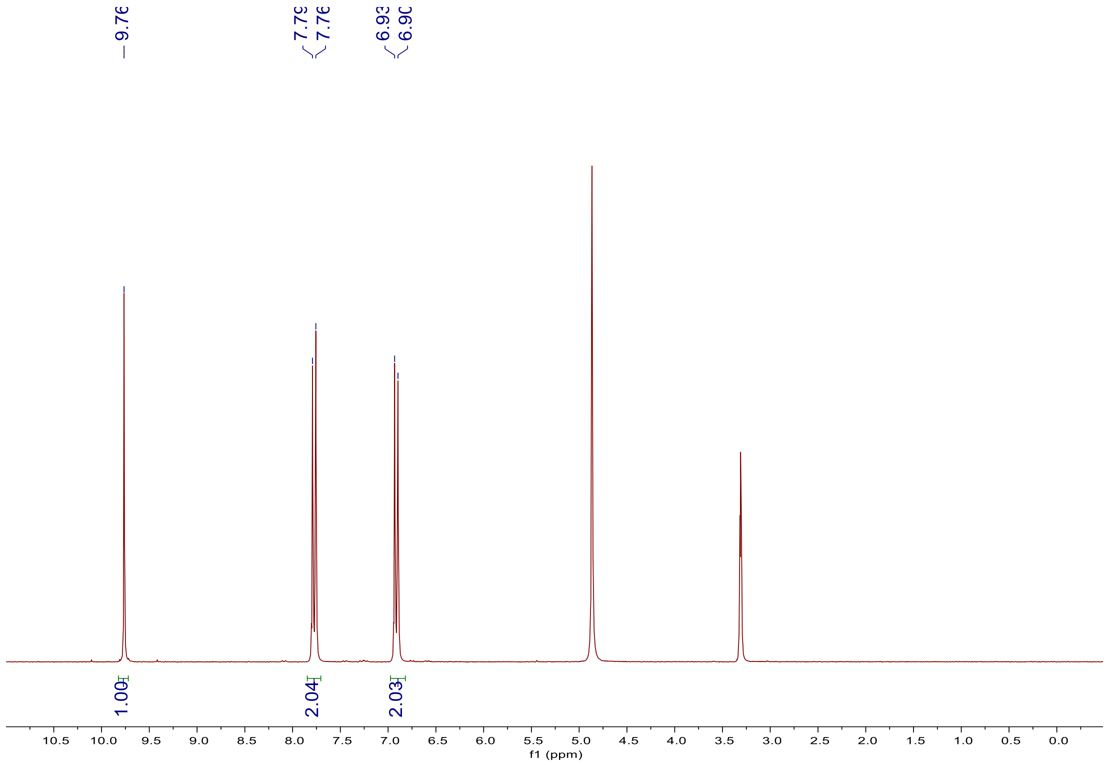

The ^1H NMR spectrum of **3** (250 MHz)

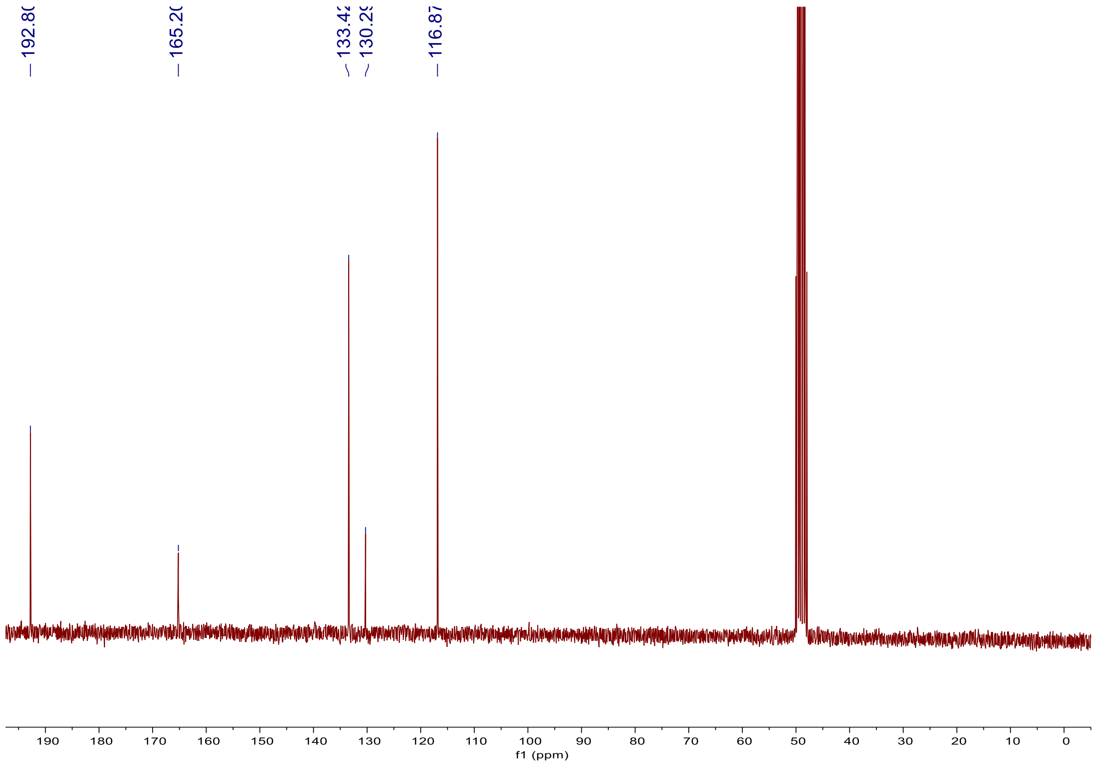

The ^{13}C NMR spectrum of **3** (62.5 MHz)

Figure S5. 1D NMR spectra of compound **4** in CD_3OD

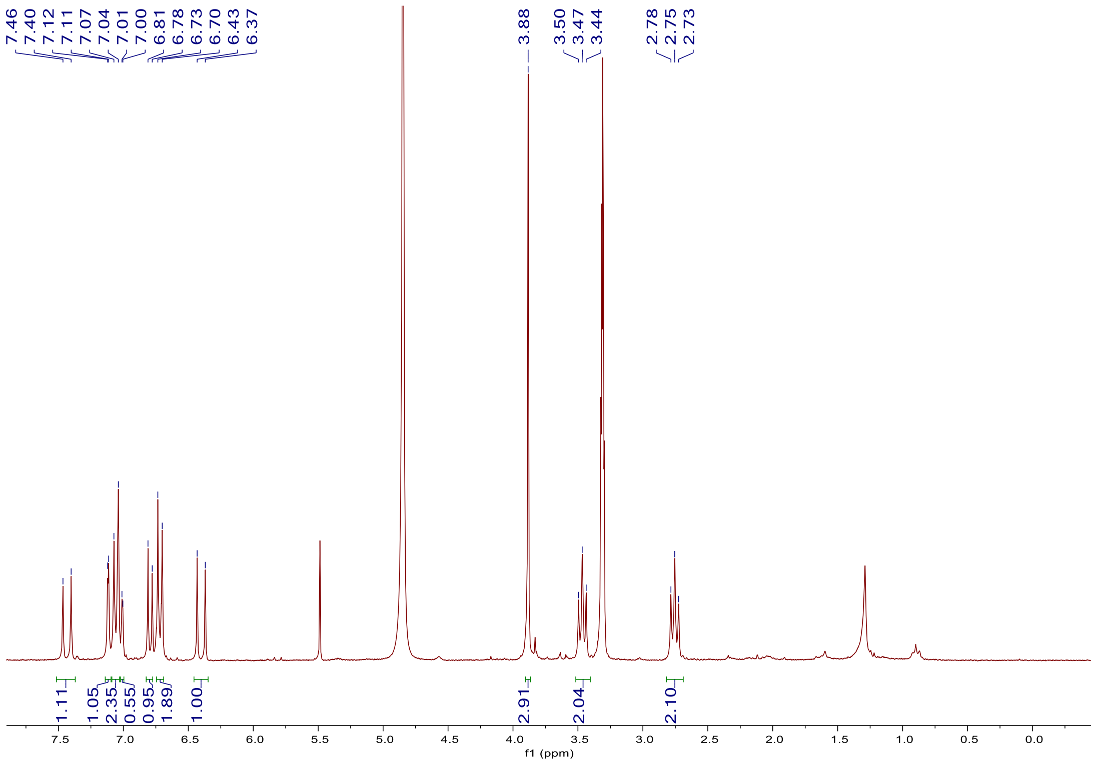

The ^{1}H NMR spectrum of **4** (250 MHz)

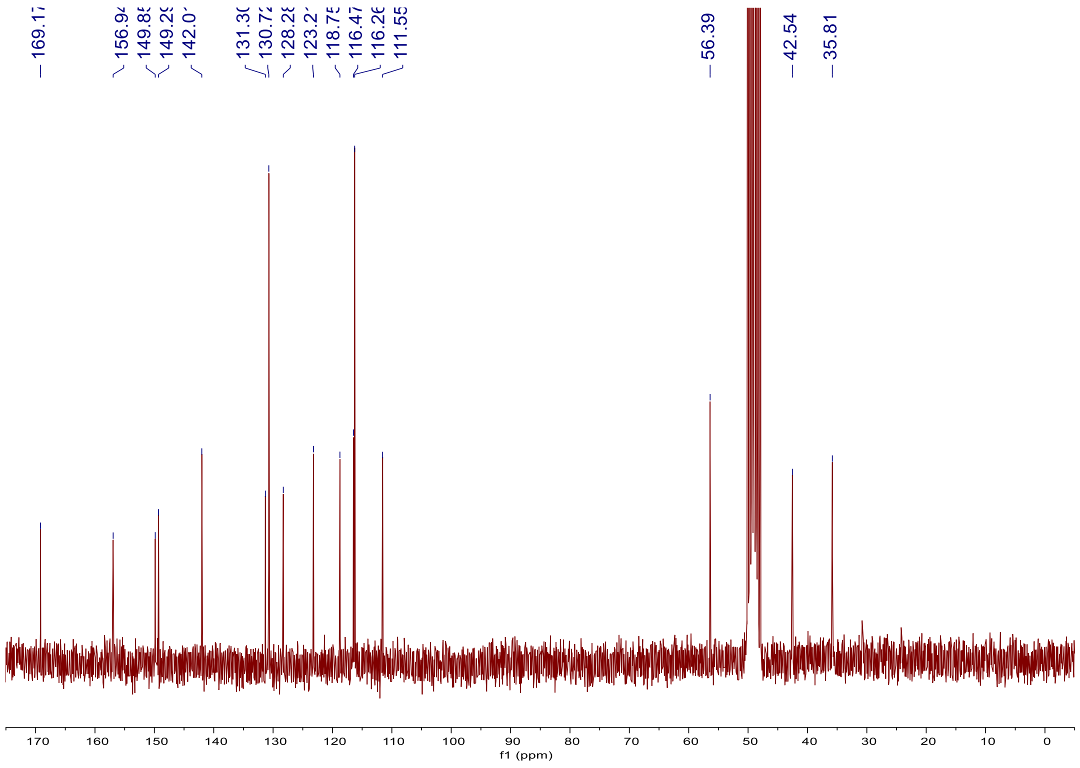

The ^{13}C NMR spectrum of **4** (62.5 MHz)

Figure S6. 1D NMR spectra of compound **5** in CD_3OD

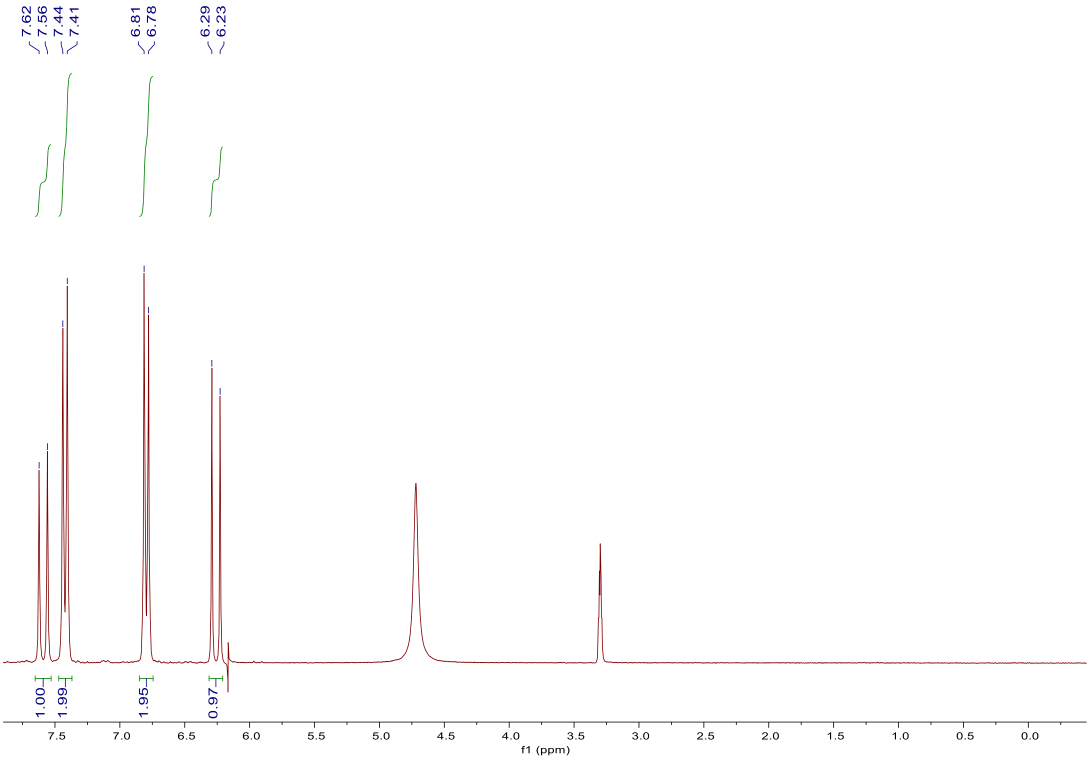

The ^1H NMR spectrum of **5** (250 MHz)

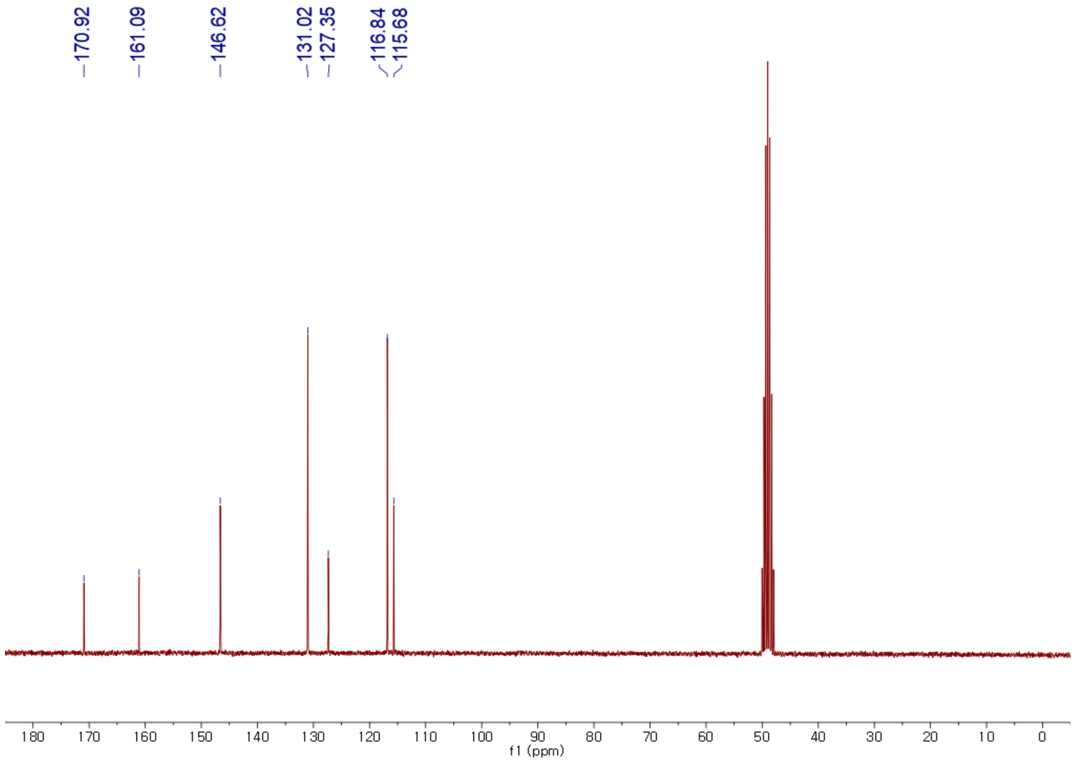

The ^{13}C NMR spectrum of **5** (62.5 MHz)
